# Supplementary material for: The genetic control of rapid genome content divergence in Arabidopsis thaliana
Source: eLife. 2026 Sep 10;14:RP108238. doi: 10.7554/eLife.108238 (PMC13561513; doi:10.7554/eLife.108238)
Supplement: Peer review history

# Editor report: eLife Assessment

**DOI:** 10.7554/eLife.108238.3.sa0

**Authors:** Detlef Weigel

**Affiliations:** Max Planck Institute for Biology Tübingen Germany

**Keywords:** Convincing, Important

This **important** study systematically investigates repeat expansion in the plant *Arabidopsis thaliana* using a new k-mer-based method, expanding on previous smaller studies, to comprehensively identify cis- and trans-acting loci associated with repeat dynamics. The approach is methodologically sound, and the exploration of different k-mer lengths and use of a more complete reference assembly strengthen confidence in the analysis while clarifying its limitations and the method is broadly applicable to large-scale short-read datasets for assessing copy-number variation and genomic repeat content. The findings are **convincing** in their scope and novelty for *A. thaliana*. It will be of interest to learn in future how the approach generalizes to species with substantially greater or lower repeat content.

# Referee report: Reviewer #1 (Public review)

**DOI:** 10.7554/eLife.108238.3.sa1

[Editors' note: this version has been assessed by the Reviewing Editor without further input from the original reviewers.]

Summary:

Overall, this study is an excellent and systematic investigation of the expansion of repeat sequences in *Arabidopsis thaliana*, and the genetic mechanisms underlying these expansions. Many of the key findings here confirm smaller studies of both repeat sequence variation and the individual genes associated with the expansion of various repeat classes. The authors present a highly effective and practical approach that requires datasets that are far more readily available than the multiple reference genomes used to annotate repeat variation in recent works. Therefore, they provide an approach that shows significant promise in non-model systems in which far less is known of repeat variation and its underlying drivers.

Strengths:

This is a very methodologically sound study that extends the relatively well-studied *Arabidopsis thaliana* repeat landscape with more systematic sampling, highlights the loci associated with repeat expansions (many of which were previously identified in a piecemeal manner), and provides some evolutionary inference on these.

Weaknesses:

Regarding cis-QTLs: I foresee at least two causes of these associations: non-repetitive cis-acting sequences that promote or permit the expansion of local repeats, and variation in repeat sequences themselves that directly tag the expanding sequence itself. It's arguable whether these are truly two distinct classes, but an attempt to discriminate between them may provide some insight as to the local factors that allow for repeat expansion, beyond the mere presence of a repeat sequence. One way to discriminate these could be to map the ~1300 12-mer frequency profiles on the reference genome, and filter any SNPs with elevated 12-mer frequency from the GWAS (or to categorize them independently).

I also have a question regarding the choice of k=12 in kmer profile analyses. Did the authors perform any GWAS with other values of K? If so, how did the results change? I would expect that as K is increased, the associations would become more specific to individual repeat families, possibly to the point where only cis-acting loci are detected. The authors show convincing evidence that k=12 is appropriate; however, I would be interested to see if/how GWAS results vary among e.g. k=10, 12, 15, 18.

# Referee report: Reviewer #2 (Public review)

**DOI:** 10.7554/eLife.108238.3.sa2

Summary:

The authors introduce a K-mer-based method for profiling repeat content within a species, applied here to 1,142 *A. thaliana* genomes sequenced with short reads. This approach allowed them to bypass the challenges of genome assembly, particularly for repetitive regions, while still quantifying copy number variation. Their analysis identified >50 trans-acting loci regulating repeat abundance, enriched for genes involved in DNA repair, replication, and methylation. They also speculate on the role of selection in shaping genome repeat content, arguing that purifying selection tends to suppress alleles that promote repeat expansion.

The work presents a scalable way to extract meaningful insights from the large quantities of short-read datasets available. However, I have several concerns regarding the methodology, scope of claims, and interpretation of results.

Strengths:

The authors leverage a large dataset, >1100 samples, of *A. thaliana*. The scale of the study is impressive and clearly bolsters their findings. Additionally, this provides a framework for future, large-scale studies and offers a solid foundation for hypothesis generation. The k-mer-based method is generally practical for large-scale analysis and should be transferable to other datasets. Finally, the authors are commendably upfront about many of the project's limitations.

Weaknesses:

The decision to use k=12 is loosely justified. While the authors performed a sweep of k-mer lengths (from 5-20) and noted computational constraints, the choice is highly dataset-specific. Benchmarking across different k values with additional datasets (especially including other species) would strengthen confidence in the robustness of the method.

All analyses rely exclusively on the TAIR10 reference genome, which is incomplete and known to collapse certain repetitive regions. This dependence raises concerns that some repeats (especially recently expanded or highly variable ones) are systematically undercounted. With improved *A. thaliana* assemblies now available, testing the method against a more complete reference would alleviate these concerns.

The manuscript's conclusions are framed in very broad terms (e.g., "shaping genome evolution in plants"). However, the study is restricted to a single species, *A. thaliana*, which may not represent other plants. While the findings may suggest general principles, the claims in the abstract and conclusion should be moderated to reflect the study system more accurately.

The identification of >50 trans-acting loci enriched for DNA repair and replication genes is compelling, but the conclusions remain correlational.

# Author comment: Author response

**DOI:** 10.7554/eLife.108238.3.sa3

**Authors:** Christopher J Fiscus, Daniel Koenig

**Affiliations:** University of California, Riverside Riverside United States; University of California, Riverside Riverside United States

The following is the authors’ response to the original reviews.

**eLife Assessment**

This important study systematically investigates repeat expansion in the plant *Arabidopsis thaliana* using a new k-mer-based method, expanding on smaller studies to more comprehensively identify cis- and trans-acting loci associated with repeat dynamics. The approach is methodologically sound and broadly applicable to large-scale short-read datasets for assessing copy number variation and genomic repeat content. While convincing in its scope and novelty, the findings would be further strengthened with exploratory analyses of datasets from other species with more or fewer repeats in their genomes.

We agree with the assessment and appreciate the Editor’s handling of our manuscript and careful consideration of our work.

**Public Reviews:**

**Reviewer #1 (Public review):**

Summary:

Overall, this study is an excellent and systematic investigation of the expansion of repeat sequences in *Arabidopsis thaliana*, and the genetic mechanisms underlying these expansions. Many of the key findings here confirm smaller studies of both repeat sequence variation and the individual genes associated with the expansion of various repeat classes. The authors present a highly effective and practical approach that requires datasets that are far more readily available than the multiple reference genomes used to annotate repeat variation in recent works. Therefore, they provide an approach that shows significant promise in non-model systems in which far less is known of repeat variation and its underlying drivers.

Thank you for your comments and careful consideration of our work.

Strengths:

This is a very methodologically sound study that extends the relatively well-studied *Arabidopsis thaliana* repeat landscape with more systematic sampling, highlights the loci associated with repeat expansions (many of which were previously identified in a piecemeal manner), and provides some evolutionary inference on these.

Weaknesses:

Regarding cis-QTLs: I foresee at least two causes of these associations: non-repetitive cis-acting sequences that promote or permit the expansion of local repeats, and variation in repeat sequences themselves that directly tag the expanding sequence itself. It's arguable whether these are truly two distinct classes, but an attempt to discriminate between them may provide some insight as to the local factors that allow for repeat expansion, beyond the mere presence of a repeat sequence. One way to discriminate these could be to map the ~1300 12-mer frequency profiles on the reference genome, and filter any SNPs with elevated 12-mer frequency from the GWAS (or to categorize them independently).

While it would be interesting to further investigate the mechanisms underlying cis-QTLs, we do not believe this dataset can distinguish between the two proposed models of cis-variation. As argued in the manuscript, the observed cis-association signals are linked to repeat-associated SNPs and therefore are most likely driven by variation in repeat copy number, supporting the latter hypothesis.

I also have a question regarding the choice of k=12 in kmer profile analyses. Did the authors perform any GWAS with other values of K? If so, how did the results change? I would expect that as K is increased, the associations would become more specific to individual repeat families, possibly to the point where only cis-acting loci are detected. The authors show convincing evidence that k=12 is appropriate; however, I would be interested to see if/how GWAS results vary among e.g. k=10, 12, 15, 18.

We attempted to regenerate the primary datasets using K = 14, but found that generating a complete 14-mer matrix for the number of samples analyzed was computationally infeasible, even after filtering low-frequency K-mers such as singletons. While this analysis could likely be done by redesigning the pipeline around a database-backed approach, we considered such development beyond the scope of this revision.

As a compromise, we regenerated the primary dataset and repeated the GWAS analyses using 10-mers. To assess the impact of K-mer length, we compare GWAS results generated from both 10-mers and 12-mers in the new Figures S19-21 and describe these results in expanded discussion on the impact of K-mer length on our results.

**Reviewer #2 (Public review):**

Summary:

The authors introduce a K-mer-based method for profiling repeat content within a species, applied here to 1,142 *A. thaliana* genomes sequenced with short reads. This approach allowed them to bypass the challenges of genome assembly, particularly for repetitive regions, while still quantifying copy number variation. Their analysis identified >50 trans-acting loci regulating repeat abundance, enriched for genes involved in DNA repair, replication, and methylation. They also speculate on the role of selection in shaping genome repeat content, arguing that purifying selection tends to suppress alleles that promote repeat expansion.

The work presents a scalable way to extract meaningful insights from the large quantities of short-read datasets available. However, I have several concerns regarding the methodology, scope of claims, and interpretation of results.

Thank you for your comments and careful consideration of our work.

Strengths:

The authors leverage a large dataset, >1100 samples, of *A. thaliana*. The scale of the study is impressive and clearly bolsters their findings. Additionally, this provides a framework for future, large-scale studies and offers a solid foundation for hypothesis generation. The k-mer-based method is generally practical for large-scale analysis and should be transferable to other datasets. Finally, the authors are commendably upfront about many of the project's limitations.

Weaknesses:

The decision to use k=12 is loosely justified. While the authors performed a sweep of k-mer lengths (from 5-20) and noted computational constraints, the choice is highly dataset-specific. Benchmarking across different k values with additional datasets (especially including other species) would strengthen confidence in the robustness of the method.

Our decision to use 12-mers to profile genome content in *A. thaliana* was based on an empirical evaluation of the sensitivity and specificity of different K-mer lengths for this application. Because our goal was to characterize intraspecific variation in genome content, we did not extend this analysis beyond *A. thaliana*. Nevertheless, we agree that alternative K-mer lengths may capture additional and potentially useful information and that using the method in other species would be interesting.

Although we found generating a 14-mer dataset to be computationally infeasible, we regenerated the primary dataset and repeated the GWAS analyses using 10-mers. To assess the impact of K-mer length, we compare GWAS results generated from both 10-mers and 12-mers in the new Figures S19-21 and describe these results in expanded discussion on the impact of K-mer length on our results.

All analyses rely exclusively on the TAIR10 reference genome, which is incomplete and known to collapse certain repetitive regions. This dependence raises concerns that some repeats (especially recently expanded or highly variable ones) are systematically undercounted. With improved *A. thaliana* assemblies now available, testing the method against a more complete reference would alleviate these concerns.

To our knowledge there is not a published gapless *A. thaliana* assembly, although several of the recent assemblies are pretty close. Although we continue to use the 1001 Genomes SNP dataset that relies on TAIR10 for the GWAS analyses, we now present Figure 2A and Figure S9 using the Col-PEK assembly (Hou, Wang, Cheng, Wang & Jiao 2022 Molecular Plant).

The manuscript's conclusions are framed in very broad terms (e.g., "shaping genome evolution in plants"). However, the study is restricted to a single species, *A. thaliana*, which may not represent other plants. While the findings may suggest general principles, the claims in the abstract and conclusion should be moderated to reflect the study system more accurately.

We concede that *A. thaliana* does not possess a typical plant genome and thus have moderated our claims throughout the paper.

The identification of >50 trans-acting loci enriched for DNA repair and replication genes is compelling, but the conclusions remain correlational.

We agree that the conclusions of our work are correlational, as they are based on GWAS associations and have not been validated through functional experiments. We would love to see tests of the hypotheses we presented, but we believe this is beyond the scope of the current study.

**Recommendations for the authors:**

**Reviewer #1 (Recommendations for the authors):**

Minor comments:

(1) The Snakemake workflow Kmer-it had a few minor bugs that prevented use with modern Snakemake versions, which I fixed as part of the review (submitted as a pull request). Please be sure to validate that the whole workflow works with the latest Snakemake version.

We appreciate the Reviewer’s testing of our software and have updated it accordingly.

(2) L162: Since you already map samples to a reference genome to filter organellar DNA, consider adding some form of filter or correction for duplication rate in paired-end data, which I have found to be one major source of batch effects as observed here between sequencing centers.

We appreciate this suggestion. In earlier versions of the analysis, we removed duplicated reads, but doing so substantially weakened the relationship shown in Figure 1C. Because it is difficult to distinguish duplicate reads arising from genuine repeat copy number variation from those resulting from technical artifacts, we ultimately chose not to include duplicate removal in the final pipeline. However, we did remove the effect of the sequencing center using a linear model.

(3) Have you used this approach with raw long-read data? Do you see any difference between Illumina and low-error-rate long read data (e.g., HiFi, R10 Nanopore with SUP calling)? This could be compared in a directly paired manner using some of the recent papers with HiFi/ONT data on 1001G accessions, e.g., Lian et al, 2024, Wlodzimierz et al, 2023, Teasdale et al, 2025. (I do not believe such a comparison is required to prove the utility of this method, but it could be of interest as such sequencing technologies become increasingly cost-competitive).

We have not evaluated this approach using raw long-read sequencing data, although we agree that it would be an interesting direction for future work. As noted in the manuscript, we detected significant batch effects attributable to sequencing center, even among datasets generated with the same sequencing technology. Given these observations, we are cautious about comparing K-mer frequencies across sequencing platforms that have distinct error profiles, as technical differences could confound biological signals.

**Reviewer #2 (Recommendations for the authors):**

(1) I would strongly suggest modulating some of the claims made in the paper, especially with regard to "genome evolution in plants", given that the paper focuses on *A. thaliana*. Alternatively, the authors could test the method in a different dataset from a different species. This would alleviate some concerns regarding the choice of k-mer length and demonstrate robustness.

We agree that *A. thaliana* is not representative of most plant genomes and have revised the manuscript to better reflect this limitation. Since the primary goal of this study was to characterize intraspecific variation in genome content within *A. thaliana*, we believe that extending the analysis to an additional species falls beyond the scope of the present work. We do not believe that our choice of K-mer length undermines the robustness of the approach. To evaluate this concern, we repeated the GWAS analyses using 10-mers and found broadly consistent results, which are presented in Supplementary Figure S19-21. These findings suggest that the major conclusions are not strongly dependent on the specific K-mer length selected.

(2) A k-mer length of 12 is quite small. There are 4^12 possible 12-mers (~17 million), so you would expect that all 12-mers would occur in the *A. thaliana* genome by chance at least once. While larger k-mers may be more computationally expensive to compute, it would be worth repeating the analysis with a larger k-mer length.

We attempted to regenerate the primary datasets using K = 14, but found that generating a complete 14-mer matrix for the number of samples analyzed was computationally infeasible, even after filtering low-frequency K-mers such as singletons. While this analysis could likely be done by redesigning the pipeline around a database-backed approach, we considered such development beyond the scope of this revision.

(3) Line 689 on page 31 should include the figure number.

Thanks! Fixed.
